# Determination of the oral health status and behaviors, treatment needs, and guardians’ perception of oral health among preschool children attending Integrated Child Developmental Scheme Anganwadi centers of Belagavi, South India: A cross-sectional study

**Published:** 2021-07-16

**Authors:** Vaibhav Kumar, Anil Ankola, Roopali Sankeshwari, Sagar Jalihal, Swarali Atre, Sreekanth Kumar Mallineni

**Affiliations:** ^1^Department of Public Health Dentistry, TPCT’s Terna Dental College, Nerul, Navi Mumbai, Maharashtra, India; ^2^Suitradhaar Strategies Pvt. Ltd, India; ^3^Department of Public Health Dentistry, KLE VK Institute of Dental Sciences, KLE Academy of Higher Education and Research, Belagavi, Karnataka, India; ^4^Department of Preventive Dental Science, College of Dentistry, Majmaah University, Al-Majmaah, Riyadh Province, Saudi Arabia

**Keywords:** rural oral health, parental perception, health services, early childhood caries

## Abstract

**Background and Aim:**

Anganwadi centers are the epicenters of health services for many Indian children. The study aims to assess the oral health status, treatment needs, and association with demographic variables, oral health behaviors, and parents’ perception among preschoolers in these Anganwadis.

**Methods:**

A descriptive cross-sectional study was conducted among 1200, 3–5-year-old preschoolers from 48 Anganwadis in Belagavi. They were examined in accordance with the WHO Oral Health Pro forma (2013). A self-administered questionnaire assessed their parents’ perception of their oral health. SPSS software (version 20) was used for statistical analysis. Chi-square test computed categorical data. One-Way ANOVA test was used for multiple group comparisons. *P*<0.05 was considered statistically significant.

**Results:**

The prevalence of dental caries was found to be 76.1% and gingival bleeding was found in 30.4% participants. The most common oral mucosal lesions were ulcers (5.1%) followed by abscess in 4.5% of children. About 67% of the parents perceived their child’s oral health as good. A staggering 98.5% of parents had not taken their children to the dentist ever. About 76.8% of the children required prompt treatment.

**Conclusion:**

Poor oral health status necessitates prompt action with age-specific targeted interventions for the curtailment of the prevalent oral maladies along with preventive strategies for the rejuvenation and resurrection of the plummeted oral health status for restoring the quality of life, coupled with motivation meted out to utilize the abundant dental services available in Belagavi.

**Relevance to Patients:**

Preschool children attending the ICDS Anganwadi centers form a nested cohort of a triangulation of need, presentation, and requirement for a targeted and focused intervention pertaining to oral health hygiene and other important constructs of overall general well-being. This hypothesis generating exploratory study opens up ways and channels for such oral health related translational activities to be planned, implemented and periodically evaluated, as part of the standard procedures and protocols of the machinery.

## 1. Introduction

India’s soul lives in its villages with nearly 65% of the population dwelling here. Despite these statistics, this population remains vulnerable in terms of literacy and access to healthcare. India’s rural literacy rate has been reported to be 73.5% compared to the urban literacy rate of 87.7%. Health literacy rates are lower in the rural population, which, along with the lower economic status, contribute to the prevailing neglect of oral health among rural Indians [1]. In-vogue 21^st^ century, a milieu of increasing health-care costs and resource constraints targeted healthcare to specific strata of society and defined age groups have become of paramount importance. However, the current system has a limited capacity to provide prompt access to dental services, particularly to young underprivileged children of rural and semi-urban areas [2].

In pursuance of the national health policy for children, India’s Government initiated the Integrated Child Developmental Scheme (ICDS). Coming under the ambit of this is an Anganwadi worker bestowed with the responsibility of organizing informed preschool education in Anganwadi centers for 3–5-year-old children. These Anganwadi workers also provide outreach services to low-income families who require immunization, healthy food, clean water, and clean toilets. An Anganwadi center forms the focal point for the delivery of these services to the children and their mothers and caters to a population of roughly 1000 people. A ”Mukhyasevika” supervises 20-–25 Anganwadi workers. Four “Mukhyasevika”-s are headed by a Child Development Projects officer (CDPO). According to official data, an estimated 1.053 million Anganwadi centers are employing 1.8 million, mostly female, workers, and helpers across the country and reach a staggering total of 58.1 million children. Today, ICDS Scheme is one of the world’s largest and most unique outreach programs for early childhood care and development. Thus, Anganwadi centers are India’s primary tool against the scourges of child malnutrition, infant mortality, and curbing preventable diseases, establishing a primary link with health and other preschool children’s services [3].

More than 90% of dental diseases are preventable. Healthy oral habits and status established during early childhood are maintained and imprinted and are of great importance for oral health and general well-being in later life [4]. The changing concept in health education is directed toward a common risk factor approach wherein the key idea is the promotion of general health by controlling risk factors that may significantly impact a lower cost, greater efficiency, and effectiveness than disease-specific approaches [5]. Child Oral Health has tremendous potential to indicating health inequalities, more pronounced during the preschool periods. Reducing these inequalities benefit wider society by potentially freeing up scarce health system resources.

Anganwadis form a nested niche for assessing preschool children coming from the lower socioeconomic backgrounds. The previous studies conducted among preschool children attending Anganwadis show the prevalence of dental caries as high as 63.58% [6]. It has been stated that the initiation of brushing habits is late among preschoolers attending Anganwadi centers, and there is a lack of awareness about brushing habits and methods among parents and Anganwadi workers [7]. In Belagavi, the prevalence rate of early childhood caries (ECC) among preschool children was 63.17% [8]. Starkly revealing, the dental health of preschool children has not been clearly documented to the same extent as school children’s oral health [9]. Therefore, these Anganwadi Centers constitute a focal point that can be easily targeted, assessed, and assisted in improving oral health and hygiene practices. Targeted interventions at the level of Anganwadis can be beneficial not only for the child but also for the parent and the Anganwadi worker. Preliminary treatment and guidance could also be provided to reduce the burden of oral diseases in these communities ultimately.

With a paucity of a documented Oral Health Survey exclusively done in Belagavi to take the Anganwadi preschoolers under its umbrella and a risk factor in the preschool age group, this study is designed with the primary aim of assessment of the oral health status and treatment needs among 3–5-year-old Children attending the Anganwadi Centers of Belagavi. The secondary aim being, assessment of the association of demographic variables, oral health behaviors, and parents’ perception of their child’s oral health status and oral hygiene practices on the dental health of these children.

## 2. Materials and Methods

A descriptive cross-sectional study was conducted among 3–5-year-old children attending Anganwadi Centers of Belagavi, Karnataka, from December 2015 to February 2016.

Permissions were obtained from the Institutional Research and Ethical Committee, CDPO, Belagavi and the in-charge Anganwadi workers.

A pilot study was done among 50 preschool children to check the comprehension, reliability of the questionnaire, the flaw, and feasibility of the intended survey and prevalence of dental caries. Using the following formula the sample size was 1135, which was rounded off to 1200.

Sample Size=4 pq/d^2^

=4×(77×23)/(2.5)^2^

=1135.

Prevalence of Dental Caries (p)=77%

Free of Dental Caries (q)=(1−p)=23%

Absolute Admissible error (d)=2.5%.

**Figure 1:**
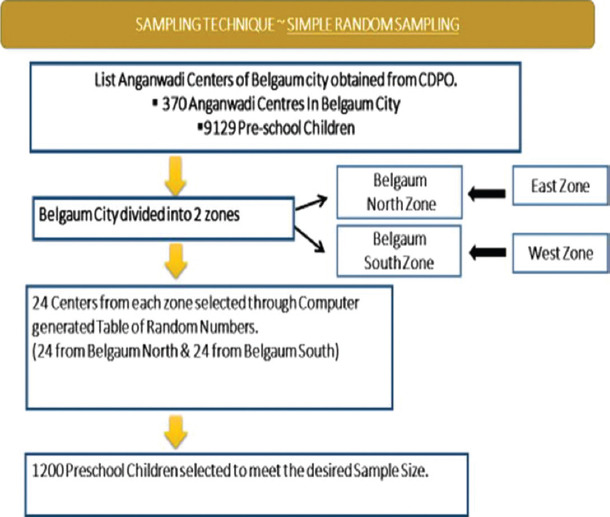
Schematic representation of sampling technique

The sampling method and procedure are depicted in Figure 1.

Written informed consent was obtained from the parents of all the participating preschool children. The inclusion criteria consisted of (1) children 3–5 years of age attending Anganwadi Centers of Belagavi; and (2) children whose parents were willing to give written informed consent. The exclusion criteria entailed the exclusion of physically or medically compromised children and children with debilitating diseases or suffering from uncontrolled systemic conditions.

On the scheduled dates, and the preschoolers’ were examined according to the WHO Oral Health Pro forma (2013) [10]. Type III examination was performed by a single trained investigator under natural light. The cotton rolls were used to clean the teeth of the food debris, and CPI probes were used to confirm doubtful dental caries. A self-administered questionnaire was prepared to assess the parents’ perception of their child’s oral health status and oral hygiene practices based on the latest WHO Questionnaire for children (2013). A minor modification was done to make the questionnaire culturally and demographically appropriate and to incorporate the indigenous oral hygiene tools used, Indian food items consumed while questions pertaining to tobacco use were omitted. The content was reviewed and validated by experts who have carried out extensive research in child and maternal health. The mean Lawshe’s Content Validity Ratio was discerned to stand at 0.92. After data collection, an interactive oral health education camp was conducted and children were also referred to a dental hospital for comprehensive treatment.

Data were analyzed using SPSS version 20 (IBM, USA). The correlation between subscales, and global health and oral health questions were evaluated using Spearman’s correlation test. Categorical data were analyzed by Chi-square test. One-Way Analysis of Variance (One-Way ANOVA) test was used for multiple group comparisons. *P*<0.05 was accepted as indicating statistical significance.

## 3. Results

**Figure 2:**
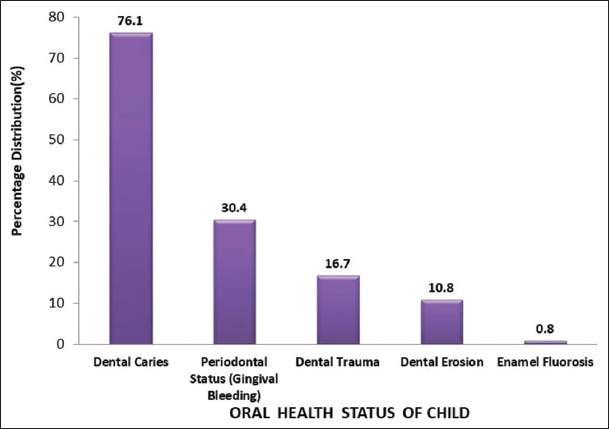
Distribution of study population according to oral health status

**Figure 3:**
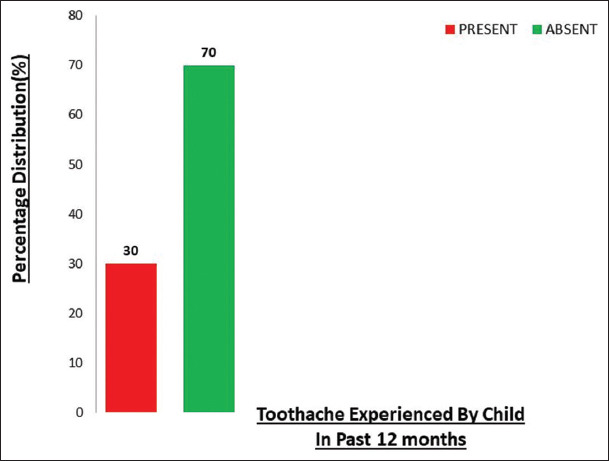
Parent’s response regarding toothache experienced by child in past 12 months

**Figure 4:**
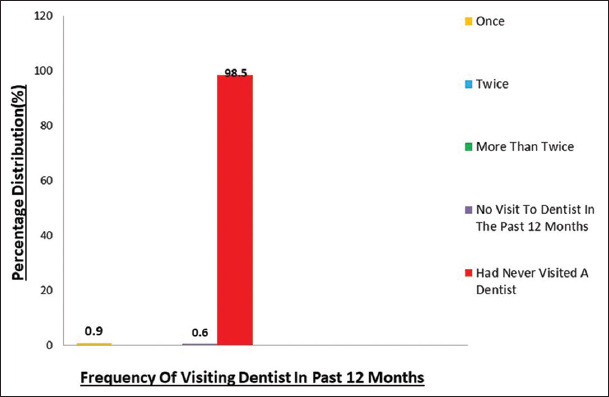
Parent’s response regarding their child’s frequency of visiting dentist in the past 12 months

The majority of the study participants were 4 years old (43.2%) and belonged to lower socioeconomic strata (64.4%). Slightly more boys (50.9%) participated than girls (49.1%). Only 6.5% of mothers are uneducated Table 1. On their examination, it was found that 76.1% of them were affected by dental caries, followed by gingival bleeding (30.4%) (Table 2 and Figure 2). Around 67% of parents perceived their child’s oral health as good. About 30% of the children had experienced toothache in the past 12 months (Figure 3). The majority (98.5%) of the parents opined that they had never taken their children to a dentist, nor had their child received any form of professional dental care (Figure 4). The marginal 1.5% of attendees had been taken to the dentist on experiencing pain/trouble with teeth, gums or mouth. Around 60% of the children cleaned their teeth themselves, and only 39.2% did so under parent’s supervision or assistance. The majority (56.9%) of children brushed their teeth once or more than once a day. All the participants used toothbrushes for cleaning their teeth and not any other oral hygiene aids. Toothpaste use was reported among around 67% of them. However, 93.4% of the parents reported being unaware that toothpaste contains fluoride.

**Table 1:** Distribution of study population according to the demographic variables

Variable	Number	Percent
Age (years)		
3	493	41.1
4	518	43.2
5	189	15.8
Gender		
Male	589	49.1
Female	611	50.9
Socio-economic status		
Upper	36	3
Upper Middle	175	14.6
Lower Middle	216	18
Lower	773	64.4
Duration child attending Anganwadi center		
1 year	505	42.1
2 years	506	42.2
3 years	189	15.8
Level of education of mother		
No formal schooling	77	6.5
Less than primary school	245	20.4
Primary school completed	322	26.8
Secondary school completed	165	13.8
High school completed	381	31.8
College/University completed	10	0.8
No female adult in the household	0	0
Total	1200	100

**Table 2:** Association of dental caries status and periodontal status with various independent variables among study subjects

Independent variable	Number of subjects	Dental caries *n* (%)	Periodontal disease *n* (%)	Oral mucosal lesions *n* (%)
Age (years)				
3	493	375 (76)	12 (2.4)*	79 (16)*
4	518	394 (76.1)	353 (68.1)*	24 (4.6)*
5	189	145 (76.7)	0*	12 (6.3)*
Gender				
Male	589	454 (77)	241 (40.9)*	19 (3.2)*
Female	611	460 (75.2)	124 (20.3)*	96 (15.7)*
Socio-economic status				
Upper	36	22 (61.1)*	12 (33.3)*	24 (66.7)*
Upper middle	175	112 (64)*	35 (20)*	12 (6.9)*
Lower middle	216	170 (78.7)*	48 (22.2)*	24 (11.1)*
Lower	773	610 (78.9)*	270 (34.9)*	55 (7.1)*
Duration of the child attending Anganwadi center				
1 year	505	399 (79)	54 (1.7)*	91 (18)*
2 years	506	367 (72.5)	251 (49.6)*	12 (2.4)*
3 years	189	140 (74.1)	60 (31.7)*	12 (6.3)*
Perception about child’s teeth				
Good	800	606 (75.8)*	305 (38.1)*	67 (8.4)*
Poor	400	308 (77)*	60 (15)*	48 (12)*
Perception of child’s gums				
Good	802	598 (74.6)	170 (21.2)*	81 (10.1)*
Poor	398	316 (79.4)	195 (52.3)*	44 (11.1)*
Teeth cleaning				
By child himself	730	589 (80.7)	212 (29)	55 (7.5)*
With parent’s assistance	470	325 (69.1)	153 (32.6)	60 (12.8)*
Frequency of teeth cleaning				
Once or more in a day	683	497 (72.8)	152 (22.3)	67 (9.8)
Less than once a day	517	417 (80.6)	213 (41.2)	48 (9.3)
Use of toothpaste				
Yes	800	606 (75.6)	212 (26.5)*	48 (12)
No	400	308 (77)	153 (38.3)*	67 (8.4)

Significantly highest prevalence of dental caries (78.9%) and gingival bleeding (34.9%) was noted among the children belonging to families with lower socioeconomic status. A significantly greater proportion of children whose parents perceived their child’s status of teeth and gums as poor had dental caries and gingival diseases than those who perceived them as good. Gingival bleeding was significantly highest among 4 years old children (68.1%) and males (40.9%). A significantly greater proportion of those who did not use toothpaste (38.3%) suffered from gingival bleeding. The Mean Caries Experience among study subjects according to the age group was illustrated in Table 3.

**Table 3:** Mean caries experience among study subjects according to the age group

Caries experience	3 Years	4 Years	5 Years	*P*-value

	Mean±SD	Mean±SD	Mean±SD	
Decayed teeth (dt)	2.89±1.42	3.01±1.85	3.32±1.90	0.012*
Missing teeth (mt)	0.09±0.04	0.21±0.09	0.09±0.03	0.421
Filled teeth (ft)	0.03±0.02	0.28±0.11	0.19±0.08	0.001*
dmft	3.01±1.38	3.50±1.84	3.60±1.72	0.001*

**Figure 5:**
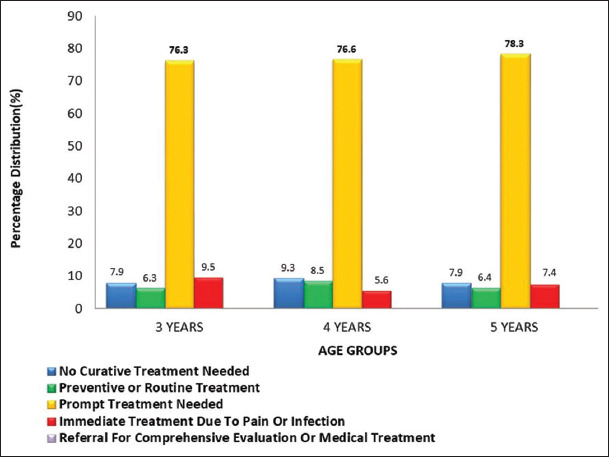
Treatment needs among different age groups

**Figure 6:**
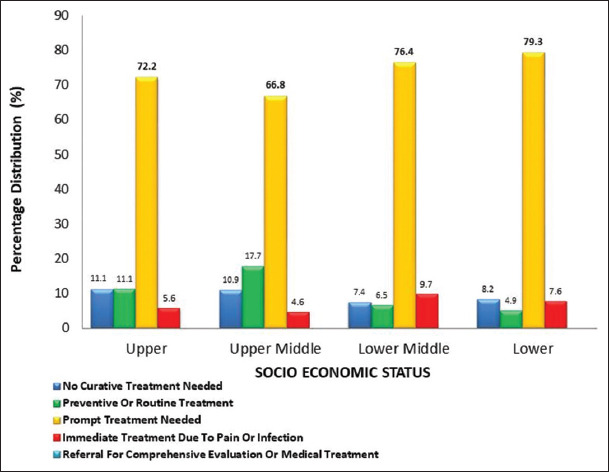
Treatment needs among various socioeconomic status

Oral mucosal lesions were observed with significantly (p<0.05) highest prevalence among 3 years age group (16%), females (15.7%), and children from upper socioeconomic status (66.7%). Children who cleaned their teeth under parent’s supervision (12.8%) had a significantly greater prevalence of oral mucosal lesions than those who cleaned their teeth themselves (7.5%) (Table 2). Enamel fluorosis occurred with significantly greater prevalence among those with upper socioeconomic status (13.8%) and those attending Anganwadi centers for 1 year (1.9%). When compared, dental erosion was found to be significantly highest among 4 years old children (20.5%), males (18%), those attending the Anganwadi centers for 2 years (18.6%), and in children from upper socioeconomic status (33.3%) than their counterparts. It was found to be in significantly greater prevalence among those whose parents perceived their oral health as good (12%) than those who perceived it as poor (9%). Dental trauma showed a significant increase with the increase in age. Just 11.8% 3 years olds suffered from dental trauma, whereas 20.5% of 4-year-old and 19% of 5-year-old children reported it. A significantly higher prevalence was observed among males (20.2%) and upper socioeconomic status children (33.3%). Among the dental trauma injuries, 6.7% of the kids reported having enamel and dentin fractures. There was a statistically significant associated report of pulp involvement in males than in females, an increased prevalence of pulp involved dental injuries with age (*P*<0.05) (Table 4). Prompt treatment was needed in >76% of the children in all age groups (Figure 5). Among those needing immediate treatment due to dental origin’s pain or infection, 9.7% belonged to the lower middle group and 5.6% belonged to the upper class, and 7.6% belonged to the lower group (Figure 6) and these findings were statistically significant (*P*=0.001).

**Table 4:** Association of enamel fluorosis, dental erosion and dental trauma with various independent variables among study subjects

Independent variable	Number of subjects	Enamel fluorosis *n* (%)	Dental erosion *n* (%)	Dental trauma *n* (%)
Age (years)				
3	493	3 (0.6)	24 (4.9)*	58 (11.8)*
4	518	3 (0.5)	106 (20.5)*	106 (20.5)*
5	189	4 (2.1)	0*	36 (19)*
Gender				
Male	589	6 (1.01)	106 (18)*	119 (20.2)*
Female	611	4 (0.7)	24 (3.9)*	81 (13.3)*
Socio-economic status				
Upper	36	5 (13.8)*	12 (33.3)*	12 (33.3)*
Upper middle	175	5 (2.9)*	12 (6.9)*	22 (12.6)*
Lower middle	216	0*	36 (16.7)*	12 (5.6)*
Lower	773	0*	70 (9.1)*	154 (19.9)*
Duration of child attending Anganwadi center				
1 year	505	10 (1.9)*	36 (7.1)*	70 (13.9)
2 years	506	0*	94 (18.6)*	94 (18.6)
3 years	189	0*	0*	36 (19)
Perception about child’s teeth				
Good	800	4 (0.5)	96 (12)*	52 (6.5)*
Poor	400	6 (1.5)	36 (9)*	148 (37)*
Perception of child’s gums				
Good	802	6 (0.7)	86 (10.7)*	180 (22.4)*
Poor	398	4 (1)	42 (10.6)*	54 (13.5)*

## 4. Discussion

The Anganwadi Centers offer basic level community healthcare through an already established, frontline primary health-care infrastructure. With a vulnerable population group and apt study setting to discern the oral cavity problems, the present status encompassed a comprehensive oral health status assessment of the subjects.

In this study, for ECC the prevalence rate of 76.1% was recorded, which was noticeably high when compared to Sonika *et al*. (48.3%) [11] and lower when compared to Al Hosani *et al*. (82–94%) [12]. Bhayade *et al*. reported an ECC prevalence of 63.58% among preschoolers attending Anganwadi Centers of Nagpur [7]. This versatile range could be attributed to single definitive diagnostic criteria not being followed, cultural differences in child-rearing, infant feeding practices, and oral health beliefs. Anganwadi children have also been reported to be suffering from malnutrition Menaker *et al.*, [13] have determined a positive association between higher caries prevalence and malnutrition. In coincidence with Hugoson *et al.*, [14] a compromised periodontal status and gingival bleeding were reported among 30.4% of the preschoolers. Gingival bleeding was significantly higher in the children belonging to the lower socioeconomic strata and greater in those children who brushed their teeth less than once a day. The prevalence of gingival bleeding in this study had a takeoff with the values reported by Dhar *et al*. [15] and Ketabi *et al*. [16], which could be because these studies have been conducted in a different study population, taking into consideration mixed dentition phases or young adolescents.

The prevalence of enamel fluorosis was 0.8%. The literature search revealed that most other studies performed on children were on mixed or permanent dentition, and there was a paucity of literature to discern enamel fluorosis exclusively for the primary dentition. According to the latest report by the Central Ground Water Board (Government of India - Ministry of Water Resources), fluoride levels found in groundwater were within allowable limits (>1.5 ml/l) in the Belagavi district. The minuscule prevalence of enamel fluorosis that has been detected could be due to including migrant population and dietary habits. Of the participants, 10.8% reported dental erosion characteristics, which could be attributed to dietary patterns. A statistically significant number of boys flinched with the occurrence of erosion, results being similar to those reported by Nayak *et al*. [17] Dental trauma was reported in 16.7% of the preschoolers with a majority (6.7%) only involving the enamel. These results fall in perspective with those documented by Chalissery *et al*. [18].

Among the Oral Mucosal Lesions, ulcers were reported to be prevalent in 5.1% and abscess in 4.5% of the participants, probably due to them suffering from viral fever-like conditions. In addition, there have been several reports of Anganwadi preschoolers suffering from malnutrition and other nutritional deficiencies [19]. Furthermore, the study duration was during the winter season, where children were reported to be more susceptible to oral ulcerations. The majority of the participants brushed their teeth once or more a day; these findings are in agreement with Al Malik *et al*. [19] study. The reason could be the urban setting of the study, with caregivers having greater knowledge and awareness regarding the importance of teeth brushing. It was observed that a more significant proportion of those who brushed their teeth less than once daily suffered from dental caries (80.6%) and gingival bleeding (41.2%) than those who brushed their teeth at least once a day. However, brushing once or more than once daily, a share of the population suffered from dental caries (72.8%) and gingival bleeding (22.3%). This could be because improper brushing technique is being performed for oral hygiene measures or failure to brush the teeth or rinse the in-between mouth meals. In this study, 60.8% of the children reportedly brushed independently. Similarly Jose *et al*. [20] reported that those who brushed by themselves reported having a greater prevalence of dental caries (80.7%) than those their parents assisted parents in cleaning their teeth. Preschool children lack adequate understanding or manual dexterity to execute oral hygiene measures, thereby requiring parental assistance or monitoring/supervision. As stated by the American Academy of Pediatric Dentistry (AAPD), a child should be supervised by adults when brushing their teeth and should be taught to spit out and not swallow the toothpaste [21]. It has also been recommended that a child should start independent brushing after attaining the age of seven [22].

A staggering 98.5% had never visited a dentist. All of those children who had to seek professional dental care (1.5%) had done so due to pain/trouble with teeth, gums, or mouth. The AAPD recommends the first visit to a dentist within 1 year of age and at least two routine visits to a dentist for a child every year [22]. Only symptomatic or painful conditions are causing severe discomfort alarm the parents to seek professional dental care. Numerous factors ranging from seemingly feeble awareness regarding dental health in the Indian scenario, fear, and anxiety associated with dental treatment to such treatment’s steep expense contribute to this. A vast majority (93.4%) of the parents seemed to be completely unaware that fluoride was an important constituent of kinds of toothpaste for caries prevention. It was revealed that the majority of the participants who required prompt treatment (including scaling) belonged to the lower or lower middle class.

The study has some limitations. The study being cross-sectional, measures the cause and effect only at a given point in time. Thus, the inability to establish a causal relationship and the persistence of temporal ambiguity contribute to certain grey areas. In addition, there are potential sources of bias, such as interviewer bias and social desirability response bias. India’s oral public health system can be strengthened by using and intertwining this existing infrastructure and well-accepted initiatives to offer targeted oral health interventions and tackle the inequitable distribution of health services and poor oral health in rural India. Dental professionals participating in such interventions should identify the disease burden, address these preschoolers’ treatment needs and engage, educate, and train the Anganwadi workers by organizing programs such as “Train the Trainer.” Anganwadi workers can disseminate basic oral hygiene methods and practices. Parents should be aware of the importance of maintaining the good health of primary teeth and thereby instill a positive attitude toward preventive and therapeutic dental aid. Following a need-based assessment, the government should allocate and release some extra funds to encapsulate the oral health and hygiene component in these Anganwadi centers and the existing health and nutritional packages offered. Oral disease prevalence among Anganwadi children should be gathered to put forth aggressive lobbying efforts for a targeted Oral Health Basic Necessity and Utility Package.

## 5. Conclusion

The oral health assessment of 3–5 year old children attending Anganwadi centers in Belagavi district revealed high prevalence of dental caries, gingival bleeding, and compromised periodontal status. A treatment needs analysis indicated the necessity of prompt treatment in majority of these children. Furthermore, poor oral hygiene habits, lack of awareness among parents about oral health and failure to seek timely help from dental professional have been found to exacerbate their oral health problems. Therefore, prompt action with age-specific targeted interventions for the curtailment of the prevalent oral maladies, preventive strategies to maintain good oral health status and motivation meted out to utilize the abundant dental services available in Belagavi is the need of the hour.
